# Changing Epidemiology and Outcomes of Hemolytic Uremic Syndrome in Children: A Prospective National Cohort Study from the Polish Pediatric HUS Registry and the Polish Registry of Renal Replacement Therapy in Children

**DOI:** 10.3390/jcm13216499

**Published:** 2024-10-30

**Authors:** Ilona Zagożdżon, Maria Szczepańska, Beata Leszczyńska, Wioleta Jarmużek, Monika Miklaszewska, Marcin Tkaczyk, Anna Medyńska, Anna Wieczorkiewicz-Płaza, Jacek Zachwieja, Piotr Protas, Paulina Rosińska, Urszula Jacher, Elżbieta Trembecka-Dubel, Danuta Zwolińska, Aleksandra Żurowska

**Affiliations:** 1 Department of Pediatrics, Nephrology and Hypertension, Medical University of Gdansk, 80-210 Gdansk, Poland; aleksandra.zurowska@gumed.edu.pl; 2 Department of Pediatrics, Faculty of Medical Sciences in Zabrze, Medical University of Silesia in Katowice, 40-055 Katowice, Poland; mszczepanska@sum.edu.pl (M.S.); etdubel@interia.pl (E.T.-D.); 3 Department of Pediatrics and Nephrology, Medical University of Warsaw, 02-091 Warsaw, Poland; beata.leszczynska@wum.edu.pl; 4 Department of Nephrology, Kidney Transplantation and Arterial Hypertension, The Children’s Memorial Health Institute, 04-730 Warsaw, Poland; w.jarmuzek@ipczd.pl; 5 Department of Pediatric Nephrology and Hypertension, Jagiellonian Univeristy Medical College, 31-007 Kraków, Poland; monika.miklaszewska@uj.edu.pl; 6 Department of Pediatrics, Immunology and Nephrology, Polish Mother’s Memorial Hospital Research Institute, 93-338 Lodz, Poland; marcin.tkaczyk@uni.lodz.pl; 7 Department of Pediatrics, Nephrology and Immunology, Medical University of Lodz, 93-338 Lodz, Poland; 8 Department and Clinic of Pediatric Nephrology, Medical University of Wroclaw, 02-091 Wroclaw, Poland; anna.medynska@umw.edu.pl (A.M.); danuta.zwolinska@umw.edu.pl (D.Z.); 9 2nd Department of Pediatrics, Department of Pediatric Nephrology, Medical University of Lublin, 20-059 Lublin, Poland; anna.plaza@umlub.pl; 10 Department of Pediatric Nephrology and Hypertension, Poznań University of Medical Sciences, 61-701 Poznan, Poland; j.zachwieja@mp.pl; 11 Department of Pediatrics and Nephrology, Medical University of Bialystok, 15-089 Bialystok, Poland; piotr.protas@umb.edu.pl; 12 Department of Nephrology, Specialist Children Hospital, 87-100 Torun, Poland; paulina.rosinska@wszz.torun.pl; 13 Department of Pediatrics, Nephrology and Dialysis, Pomeranian Medical University, 70-204 Szczecin, Poland; u.jacher@szpital-zdroje.pl

**Keywords:** hemolytic uremic syndrome, children, incidence, mortality, chronic kidney disease

## Abstract

**Background/Objectives**: Hemolytic uremic syndrome (HUS) is a known cause of acute kidney injury in children, but there are few recent reports on its epidemiology and outcome. We aimed to investigate trends in the incidence and the long-term outcomes of both Shiga toxin-producing *Escherichia coli *-HUS (STEC-HUS) and atypical HUS (aHUS) in Poland over the last 12 years (2012—2023), based on the Polish Pediatric HUS and Pediatric Renal Replacement Therapy (RRT) Registries. **Methods**: A total of 436 patients (301 with STEC-HUS and 135 with aHUS) were included. **Results**: The incidence of STEC-HUS increased during the observation period, with a mean of 3.9 cases per million age-related population (marp). The incidence of aHUS was relatively constant with a mean of 1.8/marp. The majority of patients fully recovered, although kidney sequelae were observed at 5-year follow-ups in 31% of children with STEC-HUS, 57% of aHUS subjects in the pre-eculizumab era, and 37% of aHUS subjects who had received eculizumab. The overall mortality rate was 2% for STEC-HUS and 3.7% for aHUS, with no deaths reported in children on eculizumab and mortality mainly attributed to neurological damage. A decreasing incidence of chronic kidney disease stage 5 (CKD5) due to HUS was observed. **Conclusions**: Despite an unchanging incidence of aHUS and an increasing incidence of STEC-HUS, the kidney outcomes of both diseases have improved significantly over the last 12 years. Mortality from HUS has dropped due to improved symptomatic treatment and the introduction of anti-C5 therapy. The development of CKD5 in childhood as a consequence of HUS has become exceptional.

## 1. Introduction

Hemolytic uremic syndrome (HUS) is a life-threatening, rare disease characterised by microangiopathic hemolytic anemia, thrombocytopenia, and acute kidney injury. HUS is a major cause of thrombotic microangiopathy (TMA) and can be either acquired or inherited [1]. In children, the most common cause of acquired TMA is infection-associated HUS due to Shiga toxin-producing *Escherichia coli* (STEC-HUS), which accounts for over 90% of HUS cases. In a small proportion of cases, infection-associated HUS occurs during the course of *Streptococcus pneumoniae*, the AH1N1 influenza virus, or the coronavirus COVID-19 disease. Up to 10% of TMA in children is complement-mediated (CM-HUS) due to the uncontrolled activation of the alternative pathway of the complement system [2]. Among the inherited causes of TMA in children, CM-HUS is the most common and is due to a variety of pathologic variants in complement genes and their regulatory proteins (CFH, CFI, MCP, CFB, and C3). Other inherited causes of TMA, independent of complements, include cobalamin deficiency and diacylglycerol kinase epsilon (DGKe) variants. The underlying genetic abnormality remains unidentified in up to 40% of cases [3,4,5]. Acquired CM-HUS is most commonly due to anti-complement factor H antibodies in subjects with deletions in CFHR1-3 genes [6,7]. HUS secondary to autoimmune diseases, malignancy, or following BMT or organ transplantation, as well as drug-related cases (e.g., calcineurin inhibitors, sirolimus, or cisplatin), is rare compared to its incidence in the adult population [2,8,9,10,11].

The incidence of HUS varies in different geographical regions, with an estimated incidence from 6.3 to 14.2/million age-related population/year (marp) for STEC-HUS and CM-HUS combined [12,13]. In Europe, the reported incidence of STEC-HUS is 5.6 to 7.8/marp/year, although an increasing trend has been observed in recent years [12,14]. The highest rates of 60-120/marp are noted in Argentina, mainly in children aged <5 years [15]. The incidence of CM-HUS, reported to be 0.23—2/marp/year, has been relatively constant in recent years [2,5,12,16].

The clinical outcome of HUS varies depending on the etiology of the disease. Historically, CM-HUS has been associated with a significantly worse prognosis following an acute episode, with 16–50% of patients developing stage 5 chronic kidney disease [3,5,12,17,18]. Since the introduction of anti-C5 antibody treatment (eculizumab), a targeted treatment for uncontrolled complement activation, the prognosis for CM-HUS has improved dramatically [19,20]. The prognosis for STEC-HUS patients is uncertain. About 7.3% of children require maintenance dialysis after the first manifestation of the disease. Though the vast majority recover renal function, after 12 years of follow-up, up to 51% develop chronic kidney disease [12,21,22]. The treatment of STEC-HUS remains symptomatic, but recently recommendations have been made for intensive fluid expansion in the acute phase of the disease, which may reduce thrombus formation, improve organ perfusion, and enhance the overall outcome [23,24]. The overall case fatality rate is estimated to be 1.4–3.4% for STEC-HUS in the acute phase of the disease and 6.7–15% for CM-HUS in the pre-eculizumab era [12,22,25].

This study aimed to investigate trends in the incidence of both CM- and STEC-HUS over the last 12 years (2012–2023) based on the Polish Pediatric Hemolytic Uremic Syndrome Registry and to assess early and long-term outcomes from data accumulated in both Pediatric HUS and RRT in Children Registries.

## 2. Materials and Methods

The Polish Pediatric Hemolytic Uremic Syndrome Registry, established in 2012, collects pseudo-anonymised, individual data on children treated for HUS at all twelve tertiary paediatric nephrology centres in Poland. The prospective cohort study included all patients <18 years of age receiving nephrology care for HUS in Poland between 1 January 2012 and 30 June 2023. Enrolment criteria for HUS were hemolytic non-immune induced anaemia, thrombocytopenia <150 mL, and acute kidney injury, defined according to KDIGO guidelines as a serum creatinine concentration above the upper normal limit for age or impaired urine output resulting in the need for kidney replacement therapy (KRT). Data collected included the date of the first manifestation of the disease, etiology (STEC-HUS, CM-HUS, other causes), initial symptoms, demographics, anthropometric measurements, blood pressure, modality and dates of KRT, if used, and laboratory tests, including serum creatinine, urinalysis, albumin, and urine protein excretion. Clinical data included detailed information on treatment including blood, platelet, and plasma transfusions, plasma exchange, antibiotics, and eculizumab infusions. Detailed extrarenal manifestations including neurological (seizures, coma, stroke, encephalopathy or focal neurological deficit), cardiological (hypertension, heart failure), intestinal, lung, or pancreatic involvement were also recorded. STEC infection was confirmed by the isolation of STEC from stool samples and/or the detection of Shiga toxin genes by polymerase chain reaction or the identification of the Shiga toxin antigen by enzyme immunoassay. The diagnosis of CM-HUS was made after excluding STEC infection and other known infections that can cause HUS as well as ruling out ADAMTS 13 (A Disintegrin And Metalloproteinase with ThromboSpondin type 1 motif, member 13) deficiency. CM-HUS diagnosis included complement studies (C3, CH50, anti-complement factor H auto-antibodies) and NGS genetic analysis for known pathological variants responsible for complement dysregulation. In some STEC-negative cases, the complement abnormality or pathological genetic variants were not detected, so the term aHUS is used in the study for both complement-mediated and non-complement mediated atypical HUS. Outcome data were entered annually and are presented for 1-year and 5-years of follow-up from initial manifestation. The outcome was classified as either complete recovery, defined as eGFR > 90 mL/min/1.73m^2^ and the absence of proteinuria, albuminuria, or abnormal normal blood pressure, or CKD at stage 1, stages 2–4, or stage 5, based on the calculation of eGFR using the Schwartz formula, according to KDIGO guidelines. Hypertension was diagnosed according to the European Society of Hypertension guidelines for children and adolescents as an average of at least three blood pressure measurements ≥ 95th percentile for age, sex, and height in children aged 0–15 years, or ≥140/90 mmHg for adolescents aged 16 years or older, using an appropriately sized cuff and a validated sphygmomanometer [26]. Information on deaths was collected with detailed data on the date, cause of death, and KRT status. Data on maintenance dialysis in children due to HUS were based on the Polish Registry of Renal Replacement Therapy in Children, which collects pseudo-anonymised information on demographics, the cause of chronic kidney disease stage 5 (CKD5) according to the ESPN/ERA-EDTA codification, and the date of initiation and the method of KRT, entered by nephrologists from all pediatric dialysis and pediatric kidney transplant centres in Poland. Approval for the collection and use of data for scientific purposes was obtained from the Bioethics Committee of the Medical University of Silesia for the Polish Pediatric HUS Registry (No KNW/0022/KB/262/18) and from the Medical University of Gdansk for the Polish Registry of Renal Replacement Therapy in Children (No NKBBN/280/2018). Patients and their parents or caregivers signed informed consent forms to participate in the studies. Based on the structure of the pediatric population in Poland during the study period, standardised incidence rates of aHUS and STEC-HUS and standardised incidence of CKD5 in children were calculated for the population of children in Poland from 2014 to 2022, for which complete data were available.

### 2.1. Study Population

**Figure 1:**
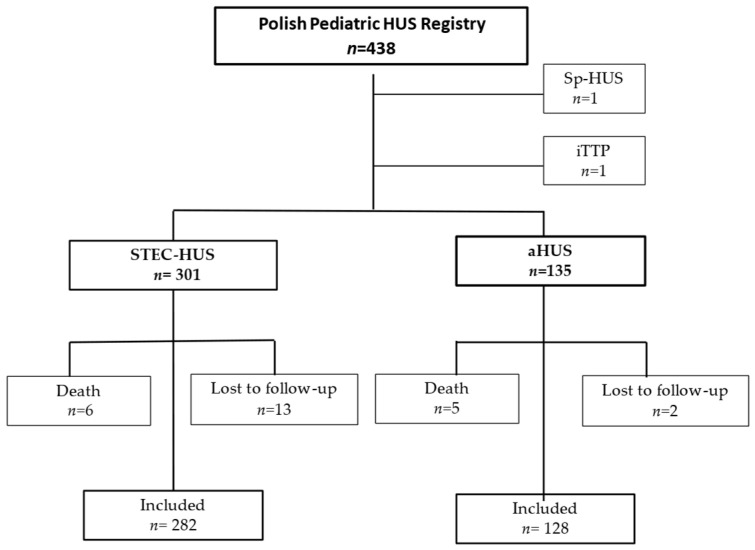
Patient selection. aHUS—atypical hemolytic uremic syndrome, STEC-HUS—Shiga toxin-producing *Escherichia coli*-associated hemolytic uremic syndrome, Sp-HUS—*Streptococcus pneumonia*-associated HUS, iTTP—immune-mediated thrombotic thrombocytopenic purpura.

Between 1 January 2012, and 30 June 2023, a total of 438 children were reported to the Polish Pediatric HUS Registry, of which 301 (68.7%) had STEC-HUS and 135 (30.8%) had atypical HUS. Two subjects, one with *Streptococcus pneumonia*-associated HUS and one with immune-mediated thrombotic thrombocytopenic purpura (iTTP), were excluded from further analysis (Figure 1).

The analysis for the aHUS cohort was performed in two groups according to the availability of eculizumab, which was reimbursed in Poland in 2018. Fifty-seven children were included in the pre-eculizumab group (treated 2012–2017) and 78 children were included in the eculizumab group (treated 2018–2022). Thirteen patients in the STEC-HUS cohort did not follow up, and six died. In the aHUS cohort, two patients did not follow up, and five died (Figure 1). All available data for those who died or were did not follow up were included in the outcome analysis. A one-year follow-up was assessed for 431 children, including 300 in the STEC-HUS group, 55 in the aHUS pre-eculizumab group, and 76 in the aHUS eculizumab group. Data for the 5-year follow-up were available for 70 children (39 with STEC-HUS, 23 with aHUS in the pre-eculizumab group, and 8 with aHUS in the eculizumab group).

### 2.2. Statistical Analysis

Continuous variables were reported as mean ± standard deviation or as median ± interquartile range and categorical variables as counts and percentages. For the comparison of continuous variables between two groups, we used a Student’s *t*-test or a Mann-Whitney U-test as appropriate, and for the categorical variables we used the chi square test. The standardised incidence rates for aHUS and STEC-HUS were calculated by direct standardisation using the World Health Organization world standard population distribution for age.

### 2.3. Outcome Analysis

The analysis of time to recovery was performed with the Kaplan-Meyer method, and the log-rank test was used to test the null hypothesis of no difference in time to the event between groups of patients. The Kendall tau coefficient was used to test for the presence of a trend in the data for incidence rates in the period between 2014 and 2022. All analyses were carried out using STATA 17.0 software (StataCorp, College Station, TX 77845, USA).

## 3. Results

The baseline characteristics data of the study population are shown in Table 1. There were no significant differences in gender distribution between the groups in the study population. The median age at the first manifestation of the disease was significantly lower in the STEC-HUS group, 2.2 years old (*interquartile range* [IQR] 1.3–4.7 years) compared to aHUS, at 3.8 years old (IQR 1.7–6.7 years). Detailed clinical presentations and laboratory findings at the first manifestation of the disease are described elsewhere.

### 3.1. Incidence

**Figure 2:**
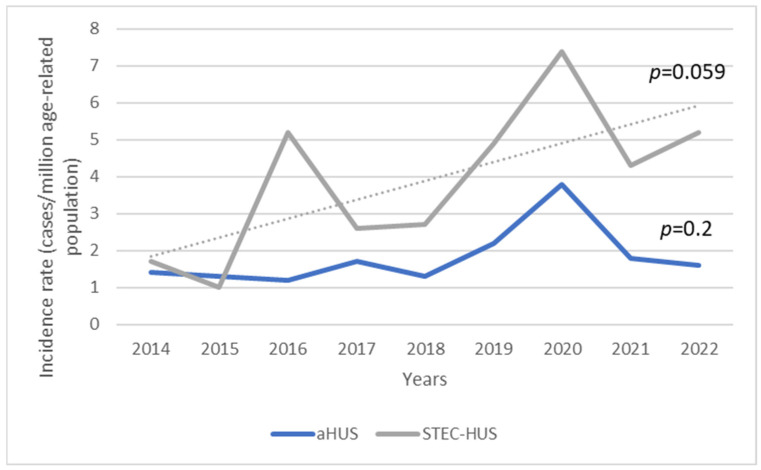
Trend in the annual incidence of Shiga toxin-producing *Escherichia coli*-associated HUS (STEC-HUS) and atypical HUS (aHUS) over the observation period.

**Figure 3:**
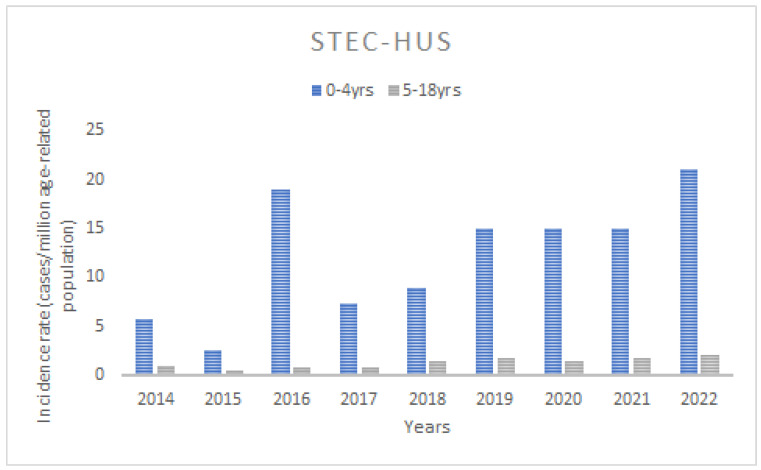
The annual incidence of the Shiga toxin-producing *Escherichia coli*-associated HUS (STEC-HUS) in the age groups of 0–4 years and 5–18 years.

**Figure 4:**
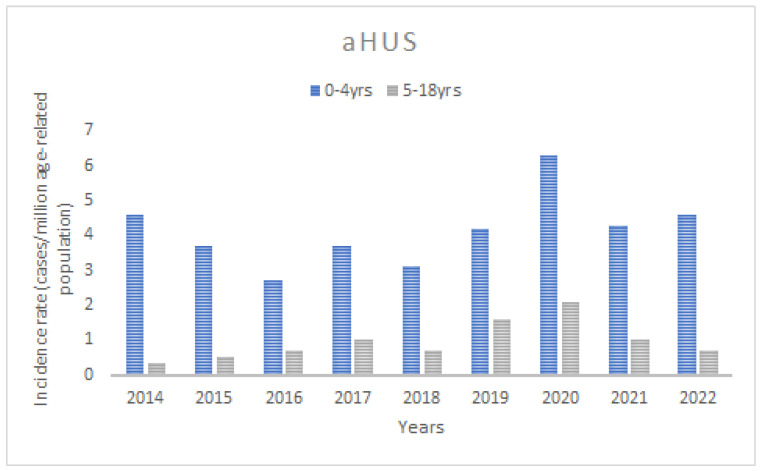
The annual incidence of atypical HUS (aHUS) in the age groups of 0–4 years and 5–18 years.

**Figure 5:**
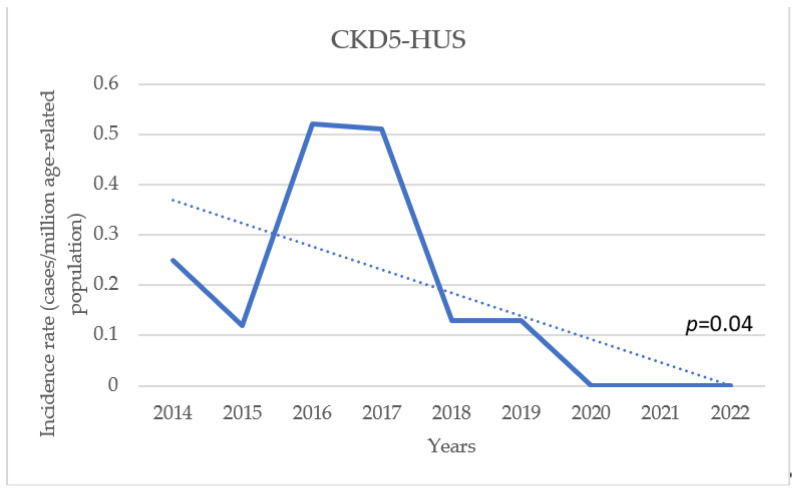
Trend in the annual incidence of chronic kidney disease stage 5 (CKD5) due to HUS (both aHUS and STEC-HUS) over the observation period.

The mean age-standardised annual incidence for STEC-HUS between 2014 and 2022 was 3.9 cases/marp (range 1.0–7.4/marp), while for aHUS it was 1.8 cases/marp (range 1.2–3.8/marp) (Figure 2). Throughout the study period, there was a trend of increasing incidence of STEC-HUS in children, at marginal statistical significance (*p* for trend 0.059), whereas there was little variability in aHUS (*p* for trend 0.2). The incidence varied significantly according to the age of the children. In the STEC-HUS group, the highest incidence was found in children under 5 years of age (the 0–4-year-old group) in each year of the analysed observation period, with an average of 12.2 cases/marp (range 2.6–21/marp) (Figure 3). The incidence was 10-fold lower for children aged 5–18 years, with a mean of 1.3/marp (range 0.5–2.0/marp). In the aHUS group, the incidence in the younger age group was also higher, with a mean of 4.1/marp (range 2.7–6.3/marp) in children aged 0–4 years, and 0.9/marp (range 0.3–2.1/marp) in those aged 5–18 years (Figure 4). The incidence of CKD5 due to HUS analysed in the same period revealed a statistically significant trend of decreasing incidence (*p* for trend 0.04) (Figure 5). The incidence of CKD5 due to STEC-HUS was 0.04 case/marp, which indicates that 1% of children with HUS-STEC developed permanent kidney damage requiring KRT. No STEC-HUS patient has started KRT in the last 5 years (2018–2022). The incidence of CKD5 due to aHUS was 0.26/marp in the pre-eculizumab era. No new cases of CKD5 due to aHUS have been reported since 2020. The percentage of children with aHUS who developed CKD5 was 14.4% in the pre-eculizumab group. Two patients in Poland started KRT after the introduction of anti-C5 treatment: one during the acute phase, for which anti-C5 treatment was not given, and one as a consequence of an episode diagnosed 5 years earlier in the pre-eculizumab era.

### 3.2. Initial Manifestation

Diarrhoea was the first symptom in the majority of children in both the STEC-HUS and aHUS groups but was statistically significantly more frequent in STEC-HUS, occurring in 95% and 50.4% of cases, respectively (Table 1). Blood in the stool as an initial symptom was observed in 18.51% of children with aHUS and 49.2% with STEC-HUS, while fever was equally frequent in both groups. Respiratory tract infection was more common in aHUS patients than in STEC-HUS patients, 34.1% vs. 10.6%, respectively. Hypertension at the disease onset occurred more frequently among the aHUS patients than the STEC-HUS patients, with rates of 60% vs. 45.8% *(p* = 0.011). At presentation, kidney replacement therapy was required for 60.7% of aHUS patients and 55.1% of STEC-HUS patients. Peritoneal dialysis was chosen more frequently for the STEC-HUS group than for the aHUS group (33.9% vs. 17.7%). In comparison, hemodialysis or hemodiafiltration was more frequently chosen for the aHUS group than for the STEC-HUS group (36.3% vs. 19.6%). A significant proportion of the children exhibited extrarenal manifestations of HUS, reported statistically more often among aHUS patients (36.2%) vs. STEC-HUS patients (19.9%) (Table 1). The most common neurological involvements were seizures, coma, stroke, encephalopathy, or focal neurological deficits, occurring in 25.1% of children with aHUS and 13.9% of children with STEC-HUS. Less common were pancreatitis, found in 18.5% of the aHUS group and 8.3% of the STEC-HUS group, and cardiac involvement, found in in 8.1% of aHUS patients and 2% of STEC-HUS patients. Since the introduction of eculizumab in Poland in 2018, the vast majority of children with aHUS (73 out of 78 patients) have been treated with anti-C5 antibodies at initial manifestation; a single patient died in the acute phase of the disease just before the introduction of eculizumab, and for the remaining four patients, no information was provided on why eculizumab was not applied.

### 3.3. Outcome

#### 3.3.1. Recovery

**Figure 6:**
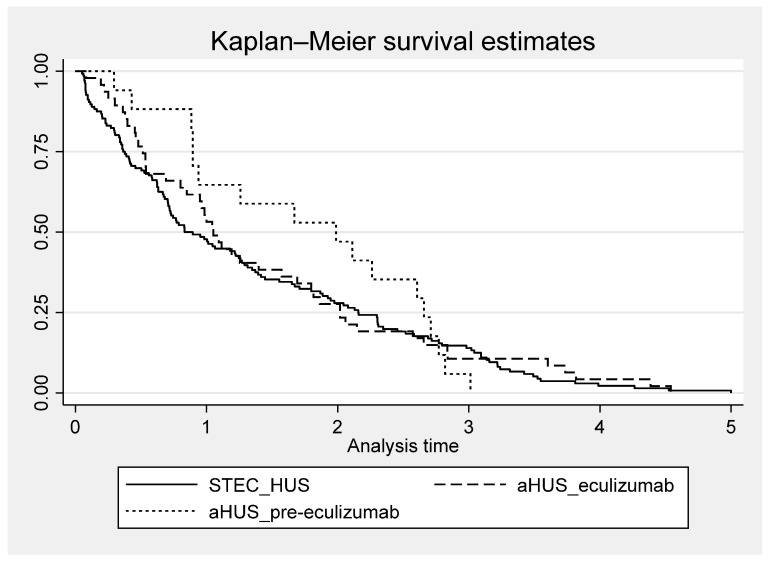
Time to recovery among those who recovered, according to diagnosis for Shiga toxin-producing *Escherichia coli*-associated HUS (STEC-HUS) and for atypical HUS (aHUS) treated in the pre-eculizumab era and eculizumab era.

After a 1-year follow-up, the majority of patients had recovered, with normal kidney function, blood pressure, and albumin/protein excretion achieved in 68% of children with STEC-HUS, 55% of those with aHUS in the pre-eculizumab group and 82% of those with aHUS in the eculizumab group. At the 5-year follow-up, 69% of patients with STEC-HUS, 43% with aHUS treated before 2018, and 63% with aHUS in the eculizumab cohort achieved full recovery. Time to recovery from the onset of the disease was longer for the aHUS pre-eculizumab cohort compared to the aHUS eculizumab cohort and the STEC-HUS group (Figure 6). Among just those who recovered, 50% of children with either STEC-HUS or aHUS in the eculizumab group achieved had full recovery by 1-year follow-up, and 75% had fully recovered by the 2-year follow-up. However, the time to recovery for the remaining 25% continued to extend up to 5 years. A total of 50% of those who recovered in the pre-eculizumab aHUS group achieved full recovery by the 2-year follow-up.

#### 3.3.2. Kidney Sequelae

**Figure 7:**
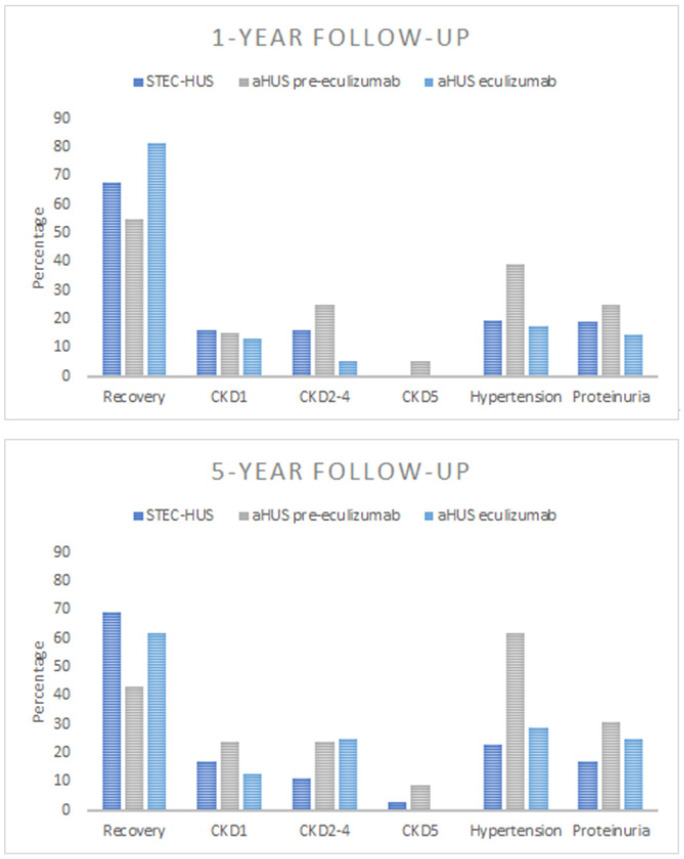
Outcomes at 1-year and 5-year follow-ups according to diagnosis for patients with Shiga toxin-producing *Escherichia coli*-associated HUS (STEC-HUS) and atypical HUS (aHUS) treated in the pre-eculizumab era and eculizumab era; CKD1—chronic kidney disease stage 1, CKD2-4—chronic kidney disease stage 2 to 4, CKD5—chronic kidney disease stage 5.

At the 1-year and 5-year follow-ups, 32% and 31% of STEC-HUS patients, respectively, exhibited kidney sequelae indicating acute kidney injury during the course of HUS. Of the STEC-HUS patients at the 1-year follow-up, 16% had CKD1, 16% developed CKD2-4, but none required kidney replacement therapy. At the 5-year follow-up in the STEC-HUS group, 17% had developed CKD1, 11% had CKD2-4, and 3% were on maintenance dialysis. For aHUS patients, outcomes varied by treatment group. At the 1-year and 5-year follow-ups, 45% and 57% of the pre-eculizumab group developed CKD: CKD1 in 15% and 24%, CKD2-4 in 25% and 24%, and CKD5 in 5% and 9%, respectively. The eculizumab group fared better at the 1-year and 5-year follow-ups, with 18% and 37% classified as CKD: 13% and 12% as CKD1, and 5% and 25% as CKD2-4, respectively. No child in the eculizumab group required maintenance kidney replacement therapy (Figure 7). After 1 year and 5 years from onset, proteinuria was present in 19% and 17% of the STEC-HUS group, 25% and 31% of aHUS patients treated in the pre-eculizumab era, and 15% and 25% of the aHUS eculizumab group, respectively. During the 5-year observation period, the percentage of hypertensive children decreased from 48.5% at onset to 23% for STEC-HUS patients, remained unchanged for the pre-eculizumab aHUS group (60% vs. 62%), and decreased from 60% to 25% for the aHUS eculizumab group. According to the Polish RRT Registry, a total of 14 patients with HUS started KRT during 2012–2022, out of 360 patients with CKD5 due to other causes. This number included 11 patients with aHUS, and 3 with STEC-HUS. Seven out of the eleven children with aHUS started maintenance dialysis within the first 6 months (the median time for the eleven patients was 0.002 years, IQR 0.002–0.42) from the initial manifestation of the disease. No child with aHUS developed CKD5 after the introduction of anti-C5 therapy. Children with STEC-HUS progressed to CKD5 at a median time of 9.8 years after onset. No child with STEC-HUS has been reported to have CKD5 since 2018. 

#### 3.3.3. Mortality

Of the 301 children with STEC-HUS, 6 died, all during the acute episode of HUS. All but one died from neurological complications such as cerebral oedema, seizures, or stroke. In one case, death was due to respiratory failure. Of the 135 patients in the aHUS cohort, 5 patients died, with three deaths in the pre-eculizumab group and two in the eculizumab group. Two children died during the acute episode of the disease and three died 0.5–2.6 years from the initial episode. One death was attributed to respiratory failure during the acute episode, and another was due to bradycardia and sudden death. Neither had received eculizumab. One patient died of unrecognized causes 6 months after the initial episode while on maintenance peritoneal dialysis after the discontinuation of eculizumab. The two remaining patients died due to the long-term consequences of the extrarenal involvement of aHUS (neurological complications and heart failure). The overall fatality rate in our cohort was 2% for STEC-HUS patients and 3.7% for aHUS patients (Table 2). The fatality rate for the aHUS pre-eculizumab group was 5.2%, compared to 2.6% for the eculizumab group. No one died while receiving active anti-C5 treatment.

## 4. Discussion

The study provides prospective population-based information on the epidemiology and outcome of HUS in childhood in Poland from 2012 to 2023, taking into account the availability of eculizumab for aHUS and the current treatment strategy for the acute phase of STEC-HUS. The average incidence of STEC-HUS in children in Poland was 3.9/marp, which is slightly lower than the reported incidence for other European countries—5.6 for the northern Italian region, and 7.8/marp for UK and Ireland [12,14]. However, it should be noted that an increase in the incidence of STEC-HUS, at marginal statistical significance, was observed in the Polish population during the study period. This trend had also been described earlier by Ardissino for children in northern Italy, specifically between 2002 and 2012. There is a lack of recent population-based national long-term studies confirming this trend in other countries. The available incidence rates typically come from regional or multicenter cohort studies rather than nationwide data. The trend observed in Poland may be attributed to gastrointestinal infections caused by strains different from those previously found in the country, changes in their virulence, or shifts in lifestyle and health behaviours. STEC is a food-borne disease that causes HUS in 4–15% of cases, with a risk of severe long-term consequences [23,27,28,29]. Previously, the most commonly identified strain, STEC O157, was the primary cause of HUS in Europe. However, it is now detected less frequently, while non-O157 strains, such as O104:H4, O103, and O55:H7 have resulted in outbreaks, as seen in Germany, Norway, and France [30,31,32]. In Poland, a higher prevalence of infections with non-O157 strains has also been observed in recent years. However, no epidemiological studies have been conducted to confirm this, highlighting the need for further investigation in a separate study. A limitation of this study is the lack of information on the serotypes of STEC, preventing a correlation with the increasing incidence and the long-term consequences of the disease. It should be noted that, despite the lack of reported serotyping, modern diagnostic methods now allow for the rapid identification of STEC in patients. This enables timely management, including fluid expansion, to prevent haemoconcentration and reduce the risk of developing HUS or minimize its consequences [23,27,33,34]. Therefore, STEC screening with strain serotyping is recommended for children with suspected infections [35].

The average incidence for aHUS during the observed period was 1.8/marp, not significantly different from that previously reported for pediatric populations, 0.5–2/marp, although it was higher than that reported by Ardissino in Italy between 2002 and 2012 [2,12]. Only a slight variation in incidence was observed in Poland during the period evaluated. It should be noted that the presented incidence rates for both STEC-HUS and aHUS are based on current available diagnostic methods—microbiological and genetic—which greatly increase the likelihood of an accurate diagnosis. In contrast, earlier studies often relied primarily on observations of clinical symptoms, in the absence of other research methods. This is particularly relevant to the calculated incidence of aHUS, since for more than half of the children with this diagnosis in our study, one of the first symptoms found was diarrhoea, including bloody diarrhoea. Historically, without the microbiological confirmation of an STEC infection, these cases could misclassified as STEC-HUS, falsely underestimating the incidence of aHUS. An essential criterion for classifying a patient as aHUS in the Polish Pediatric HUS Registry was, among other things, the exclusion of an STEC infection through polymerase chain reaction for Shiga toxin genes or Shiga toxin antigens using enzyme immunoassay.

To our knowledge, the presented study is the first study based on a nationwide registry after the introduction of eculizumab treatment with an assessment of the long-term effects of aHUS. In the presented study, similar to other studies, in all compared groups of patients—STEC-HUS, aHUS pre-eculizumab, and aHUS eculizumab, full recovery, i.e., improvement of renal function to eGFR values of >90 mL/min/1.73m^2^ in the absence of proteinuria or hypertension, was observed in the majority of patients. Still, it was less frequent in the aHUS pre-eculizumab group, especially at the 5-year follow-up. Time to recovery varied by diagnosis and treatment for aHUS, being longest for children treated before 2018 for aHUS compared to patients with STEC-HUS and aHUS treated with eculizumab after 2018. It should be noted that children with HUS, even at the CKD5 stage, who require chronic KRT can improve renal function to the extent that dialysis can be temporarily discontinued [36,37]. In children who have recovered from STEC-HUS, the dynamic evolution of eGFR is observed, and its assessment one year after the disease does not serve as a reliable predictor of long-term outcomes [38]. Although Alconcher’s observation focused solely on children with STEC-HUS, given that vascular endothelium damage in the course of aHUS occurs through the same mechanism of thrombotic changes as in STEC-HUS, it can be assumed that this observation also applies to patients with aHUS. During the long-term follow-ups in the present study, half of the patients in both the STEC-HUS and the aHUS eculizumab group who recovered achieved recovery one year after onset. In contrast, recovery in aHUS patients not treated with eculizumab was observed after approximately 2 years. It should be noted that in the subsequent years, some children continued to show improvements in renal function, as well as the resolution of proteinuria and hypertension, allowing for recovery even up to 5 years after onset (Figure 6). It is likely that the degree of renal damage, but also the renal recovery and improvement in function observed in subsequent years, may be favourably influenced by age, since both STEC-HUS and aHUS with onset in childhood mainly affect the youngest children, under 5 years of age. Similar dynamics in changes of renal function, proteinuria, and hypertension as renal sequelae were observed by Pundziene, especially in the youngest children with STEC-HUS, <1 year old, who were observed 1-, 5-, and 10-years after onset [39]. This indicates the need for the continuous monitoring of eGFR, hypertension, and proteinuria in post-HUS patients as modifiable risk factors for the progression of kidney damage. Improved prognosis for HUS is certainly influenced by ongoing clinical follow-up by a nephrologist. In the study group, only a small percentage of patients were did not follow up, while the majority continued to have regular visits to pediatric nephrology centres. (Figure 1). Thus, the applied nephroprotective treatment and good pressure control may have influenced the slowing of the progression of kidney damage, the effectiveness of which has been confirmed in other publications [40,41].

In the present study, we observed a marked decrease in the incidence of CKD5 due to HUS in recent years. No new cases of CKD5 related to STEC-HUS have been reported in the national RRT registry since 2018, and no new cases of CKD5 related to aHUS have been reported since 2020. A similar observation for STEC-HUS patients in Argentina was made by Monteverde, noting a decrease in the rate of kidney transplantation after STEC-HUS, despite the still very high incidence of the disease in the youngest child population. This means that although approximately 50–65% of patients require dialysis therapy during the acute period of the disease, and the mortality rate during this period is 1.4–3.4%, long-term outcomes are improving [21,25]. This improvement is likely due to recent changes in the symptomatic management of acute disease in STEC-HUS, including the recommendation for intensive fluid therapy, even for oligoanuric children. This approach helps prevent haemoconcentration and reduces the risk of multi-organ damage [23,33,42]. Many researchers confirmed Ake and Ardissino’s thesis that early fluid expansion decreases the risk of thrombus formation, organ ischemic damage, and central nervous system involvement. Children who received such fluid management had significantly better short-term and long-term outcomes [43,44]. Admittedly, the Polish HUS Registry does not include data on the amount of fluid administered during the acute phase of the disease. However, the observed improvement in outcomes coincides with the implementation of fluid therapy recommendations for STEC-HUS by Ardissino et al. into clinical practice. This suggests that these recommendations are likely to have positively impacted the clinical course and prognosis of the disease. A dramatic improvement in outcomes has been observed in children with aHUS since targeted treatment with an anti-C5 agent became available. Comparing the outcomes of children treated conservatively, prior to the availability of eculizumab, with those who received the drug, it is clear that chronic kidney disease, in some cases requiring KRT, as well as hypertension and proteinuria, were found significantly more often in the historical cohort (Figure 6). Early use of targeted treatment can minimize the effects of the disease and improve long-term outcomes. For selected patients, it is possible to discontinue chronic prophylactic treatment with an anti C5 agent, which has been confirmed by many observations. However, patients should remain under continuous nephrology care to monitor for possible relapses [4,7,45]. It should be emphasized that in the historical group of children with aHUS treated before 2018, who required maintenance dialysis, permanent loss of kidney function was observed in 7 out of the 11 children during the first days or weeks after onset. Hence, the careful monitoring of disease activity is advisable to allow prompt initiation of treatment and avoid chronic sequelae in case of relapse.

Despite the observed improvement in the prognosis for children with HUS, it should be emphasized that it remains a life-threatening disease. During the acute phase, it is primarily associated with a risk of death, often due to central nervous system involvement. In the study group, 6 deaths were recorded among children with STEC-HUS, all during the acute episode, and 5 deaths were recorded among children with aHUS, 2 of them during the acute phase, while there were no deaths among patients actively treated with eculizumab.

## 5. Conclusions

The incidence of STEC-HUS in children has increased in Poland over the last decade; however, the long-term outcome has improved, with a decreasing number of children with permanent kidney damage being observed, probably due to the better intensive symptomatic treatment given during the acute phase.

The incidence of aHUS is relatively constant. Following the introduction of eculizumab for the treatment of aHUS, the long-term prognosis has improved significantly. No patients receiving anti-C5 antibodies have required maintenance kidney replacement therapy to date, and fewer patients have developed hypertension, proteinuria, or chronic kidney disease.

Despite the observed improving outcomes, HUS remains a life-threatening condition, with mortality mainly in the acute phase of the disease due to neurological complications.

The majority of children with HUS, both STEC-HUS and aHUS, recovered during the observation period, but some of them achieved full recovery even 5 years after onset, suggesting that their kidney status should be systematically followed.

## Figures and Tables

**Table 1:** Baseline characteristic of study population.

	aHUS (*n* = 135)	STEC-HUS (*n* = 301)	*p*-Value
Gender Female Male	67 (49.6%) 68 (50.4%)	164 (54.5%) 137 (45.5%)	0.401
Age at Onset, Years (IQR)	3.8 (1.7 -6.7)	2.2 (1.3-4.7)	0.002
Initial Symptoms Diarrhoea Blood in stool Respiratory tract infection Fever	70 (51.8%) 25 (18.5%) 46 (34.1%) 61 (45.2%)	286 (95%) 148 (49.2%) 32 (10.6%) 121 (40.2%)	<0.001
Hypertension at Onset	81 (60%)	137 (45.8%)	0.011
KRT at Onset	82 (60.7%)	166 (55.2%)	0.414
KRT Modality at Onset			<0.001
Peritoneal dialysis	24 (17.8%)	102 (33.9%)	
Hemodialysis/hemodiafiltration	49 (36.3%)	59 (19.6%)	
PD and HD/HDF	8 (5.9%)	6 (2%)	
Extrarenal Manifestation Neurological Pancreatitis Cardiac involvement	49 (36.2%) 35 (25.9%) 25 (18.5%) 11 (8.1%)	60 (19.9%) 42(13.9%) 25 (8.3%) 6 (2%)	0.004
Eculizumab Treatment at Onset	73 (54%) *	2 (0.7%)	0.001

* Eculizumab has been available in Poland since 2018. aHUS—atypical hemolytic uremic syndrome, STEC-HUS—Shiga toxin-producing *Escherichia coli*-associated hemolytic uremic syndrome, IQR—interquartile, KRT—kidney replacement therapy, PD—peritoneal dialysis, HD—hemodialysis, HDF—hemodiafiltration.

**Table 2:** Mortality and causes of death in aHUS and STEC-HUS patients.

	aHUS	STEC-HUS	*p*-Value
**Mortality Rate**	5/135 (3.7%)	6/301 (2%)	0.29
**Cause of Death**			0.13
Cardiac arrest/sudden death	2 (40%)	0	
Heart failure	1 (20%)	0	
Respiratory failure	1 (20%)	1 (16.6%)	
Neurological complications	1 (20%)	5 (83.3%)	
**Death during Acute Episode of HUS**	2 (40%)	6 (100%)	0.06
**Death According to KRT Modality** PD CVVHDF No KRT	1 (20%) 0 4 (80%)	2 (33.3%) 3 (50%) 1 (16.6%)	0.08

aHUS—atypical hemolytic uremic syndrome, STEC-HUS—Shiga toxin-producing *Escherichia coli*-associated hemolytic uremic syndrome, KRT—kidney replacement therapy, PD—peritoneal dialysis, CVVHDF—continuous veno-venous hemodiafiltration.

## Data Availability

Restrictions apply to the availability of these data. Data were obtained from the Polish Society of Pediatric Nephrology and are available from the authors with the permission of the Polish Society of Pediatric Nephrology.
